# Retrospective study of pathogens involved in vaginitis among Chinese women

**DOI:** 10.1186/s12905-023-02510-0

**Published:** 2023-07-08

**Authors:** Zhengmei Pan, Yanzhi Wu, Yan Li, Xi Hu, Yiyue Zhao, Yang Liu

**Affiliations:** Department of Reproduction, the Second Affiliated Hospital of Kunming Medical University, No. 374 Dianmian Avenue, Wuhua District, Kunming, 650000 Yunnan China

**Keywords:** Inflammation, Mixed infection, Single infection, Vaginal infection

## Abstract

**Background:**

To explore the pathogen distribution in Chinese females with vaginitis.

**Methods:**

This retrospective study included Chinese females with vaginitis admitted at the outpatient department of the Gynecology Clinic of the Second Affiliated Hospital of Kunming Medical University between January 2013 and June 2013. Data on the vaginal pathogens and inflammation were analyzed.

**Results:**

The vaginal secretions from 15,601 gynecologic outpatients were abnormal, including 8547 (54.78%) with vaginal infection and 7054 (45.22%) without. In patients with vaginal infections, a single infection was observed in 69.72% (5959/8547) of them, and mixed infection was observed in 30.28% (2588/8547). The differences in age and inflammation grade between the infection and no-infection groups were statistically significant (all *P* < 0.001). In addition, multiple types of vaginitis could be diagnosed in patients with mixed infections.

**Conclusions:**

About half of the Chinese women with abnormal vaginal secretions are positive for pathogens in the study period. Patients’ age and inflammation grade are associated with co-infection. From the public health perspective, this study suggests that the importance of vaginal hygiene should be enforced in Chinese women.

## Background

Vaginitis is the inflammation of the vaginal mucosa and submucous connective tissues and is one of the most common gynecological diseases. The vagina can be easily infected by various pathogenic microorganisms because of its anatomical and physiological characteristics. Many exogenous and opportunistic pathogens can cause vaginitis. Vaginitis is characterized by pruritus and burning vulvar pain and can even lead to infertility. The prevalence of vaginitis is around 11%-20% in women [1]. The prevalence varies widely among regions in China, from 5.9% to 51.6% [2–10]. The risk factors for vaginitis are age, having more than one sexual partner, lower use of barrier contraceptives, menstruation, abortion, and poor hygiene [10, 11].

Vaginitis can be divided into bacterial vaginosis (BV), vulvovaginal candidiasis (VVC), aerobic vaginitis (AV), *Trichomonas* vaginitis (TV), cytolytic vaginosis (CV), and desquamative vaginitis (DV). The most common types of vaginitis in the clinic are BV, VVC, AV, and TV. The prevalence of BV is the highest (51%), followed by VVC (6.5%) and TV (2.5%) [7–10, 12, 13]. AV, BV, and TV are associated with pruritis and changes in the color, consistency, and odor of the vaginal secretions [14–16]. The normal pH of the vagina is 4.0, and vaginitis is associated with an increase in the pH and an amine odor [17]. The infection is asymptomatic in the early stages, and symptoms severity increases in the later stages. This lag in symptoms can increase treatment complexity, aggravate the patients’ psychological and financial burden and significantly impact the patient’s physical and mental health. Vaginitis during pregnancy can adversely affect perinatal outcomes [18]. Therefore, there is a need to improve the understanding of vaginitis and identify the infection type and pathogenic microorganisms to provide the appropriate treatments. A better understanding could help improve the success rate of the treatment of vaginitis.

The most common method for diagnosing vaginitis is a microscopic examination under phase contrast microscopes. *Lactobacillus* grade, number of leukocytes, the proportion of toxic leukocytes, background flora, and the proportion of parabasal epitheliocytes can also be used for a more accurate diagnosis of vaginitis [19, 20]. Another method is the cloning and sequencing of the 16S ribosomal RNA genes [21]. Nevertheless, this method is not widely used due to its high cost. Diagnostic kits can also be used to measure the levels of five indicators: (a) hydrogen peroxide (H_2_O_2_) concentration to assess the abundance of probiotic *Lactobacilli*; (b) leukocyte esterase (LE) activity to measure the presence of inflammation in relation to the predominating bacterial morphotypes in the vagina [22]; (c) sialidase activity to measure the presence of BV-associated bacteria such as *Gardnerella vaginalis* and *Prevotella bivia* [15]; (d) β-glucuronidase (Gus) activity, which is related to *Escherichia coli* and Group B *Streptococcus*; (e) coagulase activity to check for the presence of *Staphylococcus aureus* [14]. Such kits do not require special equipment or materials, the results are objective, and the kits have good sensitivity and specificity.

In China, the current status of diagnosis and treatment of lower genital tract infection is characterized by a high cost, high recurrence rate, low cure rate, and poor rational use rate of antibiotics. The treatment of patients by gynecologists without identifying the pathogenic microorganisms leads to poor therapeutic effects, recurrences, and long disease courses, which can have serious consequences, especially for young women with wishes for reproduction.

Hence, this large cross-sectional study aimed to examine pathogenic bacteria distribution in Chinese females with vaginitis. The results should help improve the understanding, correct diagnosis, treatment, and prognosis of vaginal infections in Chinese women.

## Methods

### Study design and population

This retrospective study included Chinese females with vaginitis admitted at the outpatient department of the Gynecology Clinic of the Second Affiliated Hospital of Kunming Medical University between January 2013 and June 2013.

Women diagnosed with vaginitis were included. The exclusion criteria were (1) diabetes, (2) took broad-spectrum antibiotics in the last month, (3) using immunosuppressive agents or corticosteroids for more than 1 month, (4) suffering from severe infectious diseases, (5) vaginal administration or sexual intercourse in the last 24 h before the examination, or (6) incomplete data. Vaginitis diagnosis included VVC (spores or hyphae of *Monilia* were seen under the microscope), TV (*Trichomonas* were seen under the microscope by the hanging drop method), AV (leukocyte esterase was positive, accompanied by β-glucuronidase positive and/or coagulase positive), BV (leucocyte esterase was positive, accompanied by clue cell positive and/or sialidase positive), and other types of vaginitis (other types of single bacteria were seen under the microscope, except for BV, VVC, TV, and AV).

This study was approved by the ethics committee of the Second Affiliated Hospital of Kunming Medical University. All procedures were performed according to the tenets of the 1964 Helsinki Declaration and its later amendments or comparable ethical standards. The requirement for informed consent was waived due to the retrospective design.

### Data collection and definition

The patients were kept in the lithotomy position, fully exposing the vagina with a disposable speculum to sample the secretions at the lateral vaginal wall and posterior fornix using a cotton swab. If the patient had no sexual history, the secretions were sampled 4 cm inside the orificium vaginae. Two samples were taken from each patient, one for VVC, TV, and inflammation examination under the microscope (Olympus CX23, Japan) and the other for the AV/BV Five Joint Assay Kit (ABV, Beijing ZhongSheng JinYu Diagnostic Technology Co., Ltd., China; https://patents.google.com/patent/CN101792791A/en). All patients were examined by professional gynecologists.

The grade of vaginitis was according to *Non-gestational vaginitis, American College of Obstetricians and Gynecologists, 2020* and *Guidelines for the diagnosis and treatment of bacterial vaginosis in China and the United States, 2021*, including: grade I, many bacilli vaginalis, the visual field was full of epithelial cells, 0–5 pyocytes or white blood cells per high power field (HPF); grade II, a few bacilli vaginalis, half of the visual field was full of epithelial cells, 6–15 pyocytes or white blood cells per HPF; grade III, a few bacilli vaginalis, and only a few epithelial cells in the visual field, 16–30 pyocytes or white blood cells per HPF; grade IV, no bacilli vaginalis, no epithelial cells in the visual field, > 30 pyocytes or white blood cells per HPF. Grades I and II were considered normal, while grades III and IV were considered abnormal.

### Statistical analysis

SPSS 17.0 (SPSS Inc., Chicago, IL, USA) was used for statistical analysis. Categorical variables were expressed as n (%). The chi-square test was used for comparison among groups. *P* < 0.05 was considered statistically significant.

## Results

The vaginal secretions from 15,601 gynecologic outpatients were examined and were abnormal, including 8547 (54.78%) with vaginal infection and 7054 (45.22%) without. In patients with vaginal infections, a single infection was diagnosed in 69.72% (5959/8547) and mixed infection in 30.28% (2588/8547). The differences in age distribution between the vaginal infection and no vaginal infection groups were statistically significant (*P* < 0.001) (Table 1).

**Table 1:** Basic characteristics

Variable, n (%)	All (*n* = 15,601)	Infection (*n* = 8547)		No infection (*n* = 7054)	*P*
		Single infection (*n* = 5959)	Mixed infection (*n* = 2588)		
Age (years)					< 0.001
≤ 20	981 (6.29%)	379 (38.63%)		602 (61.37%)	
21–30	4519 (28.97%)	3049 (67.47%)		1470 (32.53%)	
31–40	5143 (32.96%)	2970 (57.75%)		2173 (42.25%)	
41–50	3601 (23.08%)	1721 (47.79%)		1880 (52.21%)	
> 50	1357 (8.70%)	428 (31.54%)		929 (68.46%)	
Inflammation grade					< 0.001
II	3217 (20.62%)	48 (1.49%)	0	3169 (98.51%)	
III	9079 (58.20%)	5010 (55.18%)	1166 (12.84%)	2903 (31.97%)	
IV	3305 (21.18%)	901 (27.26%)	1422 (43.03%)	982 (29.71%)	

*AV* aerobic vaginitis, *BV* bacterial vaginosis, *TV* trichomonas vaginitis, *VVC* vulvovaginal candidiasis

**Fig. 1:**
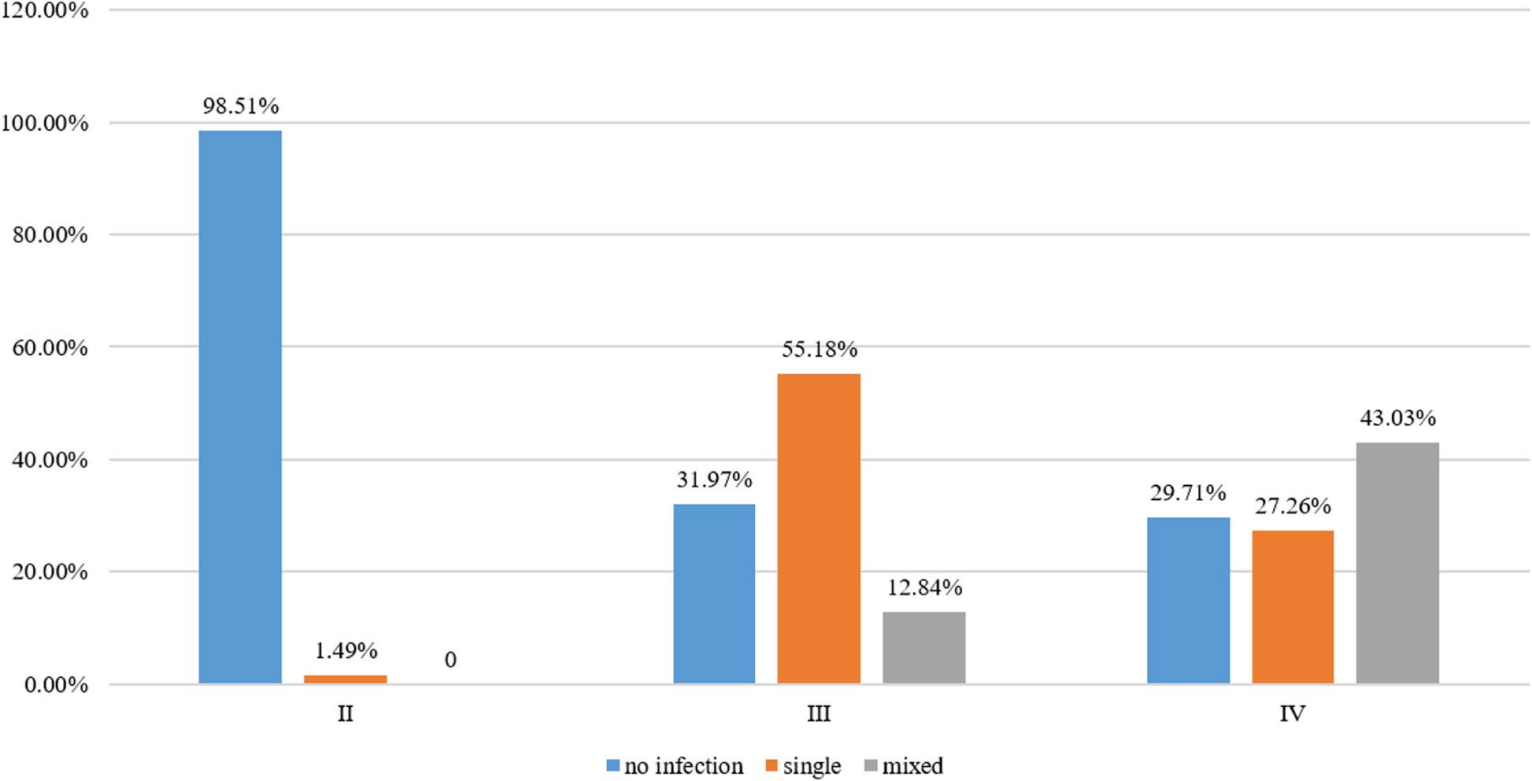
Inflammation grading distribution among no infection, single infection, and mixed infection

**Fig. 2:**
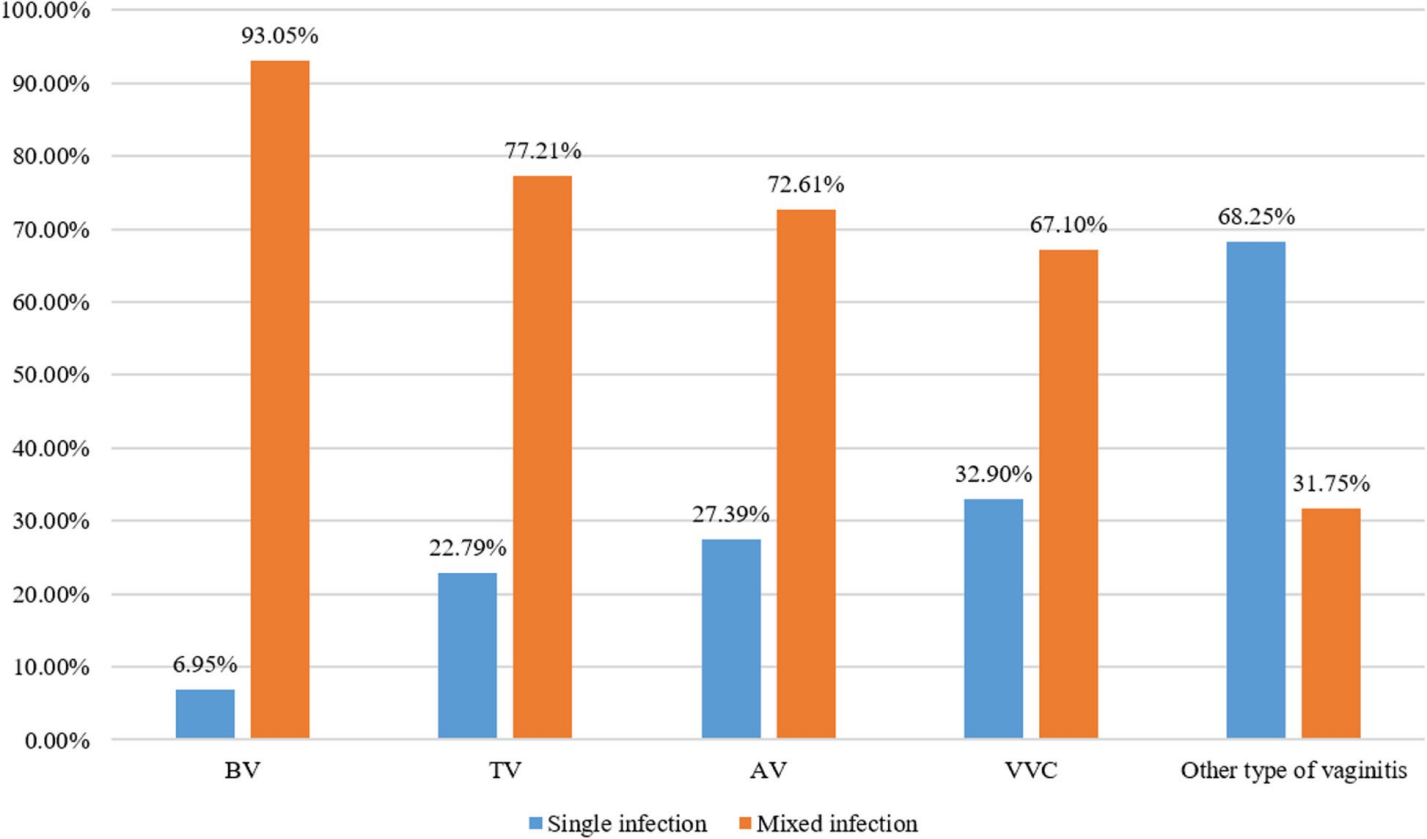
Vaginitis diagnosis distribution single infection, and mixed infection

Inflammation grades II, III, and IV were observed in 3217 (20.62%), 9079 (58.20%), and 3305 (21.18) patients, respectively (Table 1). Women with no infection (*n* = 7054) showed grade II, III, and IV inflammation in 3169 (44.92%), 2903 (41.15%), and 982 (13.92%) patients, respectively. The women with single infection (*n* = 5959) showed grade II, III, and IV inflammation in 48 (0.81%), 5010 (84.07%), and 901 (15.12%) patients, respectively. The women with mixed infection (*n* = 2588) showed grade II, III, and IV inflammation in 0, 1166 (45.05%), and 1422 (54.95%) patients, respectively (Table 1 and Fig. 1). The differences in the inflammation grade distribution between the infection and np-infection groups were statistically significant (*P* < 0.001) (Table 1). In addition, mixed infection was found in 92.29% (*n* = 1125) of the 1219 patients with BV, 77.21 (*n* = 105) of the 136 patients with TV, 72.61% (*n* = 1365) of the 1880 patients with AV, 67.21% (*n* = 1240) of the 1848 patients with VVC, and 31.75% (*n* = 2192) of the 6903 patients with other types of vaginitis (Fig. 2).

## Discussion

In this study, pathogenic bacteria were detected in 54.78% of the women with vaginitis. Single and mixed infections were found in 69.72% (5959/8547) and 30.28% (2588/8547) of all infections, respectively. Among all patients, 79% (12,384/15,601) had grade III-IV inflammation, out of which 47.73% (5911/12,384) had single infections, while mixed infection was present in 20.90% (2588/12,384) of the cases. This study suggests that the importance of vaginal hygiene should be improved in Chinese women.

Dai et al. [2] reported that BV was the most common cause of vaginitis among Tibetan women, followed by VVC and TV. In Shanghai, among women with vaginal symptoms, the positive pathogen rate was 65.6%, and TV accounted for 18.9% of the patients; mixed infections were found in 35.0% [8]. Zhang et al. [23] showed that 65.5% had a single infection among patients with AV. In this study, mixed bacteria had the highest detection rate in the single infection, accounting for 79.06% of all infection cases. Mixed bacteria refer to a non-specific infection that cannot be clearly classified but can cause vaginal mucositis. Therefore, mixed bacteria need to be actively treated to avoid further aggravation and the occurrence of infection. Previous studies investigating vaginitis in China also showed a high prevalence of mixed infections [8, 24]. The epidemiology of vaginitis depends upon several factors, including socioeconomic factors, hygiene, the environment, sexual practices, and lifestyle habits, among others. Therefore, comparisons among countries and areas must be made with caution.

Changes in the local microenvironment after infection result in the proliferation or replication of conditional pathogens, and various types of vaginitis can combine, leading to mixed infection. In this study, the most common type of mixed infection was a double infection, accounting for 69.05% (1787/2588), and patients with BV + mixed bacteria accounted for a large proportion (22.30%; 577 out of 2588 patients). The analysis demonstrated that normal *Lactobacillus* in the vagina of BV patients was reduced while the anaerobes multiplied in large numbers. Besides, amine substances are produced, and the pH of the vagina increases, resulting in the loss of the normal defense function. In previous studies conducted in China and India, mixed infection with AV and BV had a high incidence [2–10, 25]. BV can lead to pelvic inflammation, postpartum endometriosis, amniotic fluid infection, chorioamnionitis, and premature delivery [1, 26]. Thus, both single and mixed infections of the genital tract should be treated as early as possible. *Lactobacillus* has been shown to account for > 95% of all permanent bacteria in the vagina and plays an important role in maintaining the vaginal microecological balance [27, 28]. There were 15,231 (97.63%) patients with abnormal vaginal secretions in this study, indicating that most of the subjects were in a dysfunctional state of vaginal microecology despite pathogens not being detected in the vaginal secretions using traditional methods.

The incidence of vaginitis is closely related to age. Studies have shown that women of different ages have different levels of vaginal infections due to their diverse physical conditions and personal habits [29, 30]. The leading cause of vaginal infection in women of childbearing age is sex. Improper contraceptive measures or sexual behavior can lead to vaginal infection. The incidence of vaginal infection was 67.47% (3049/4519) in the 21–30 age group and 57.75% (2970/5143) in the 31–40 age group in this study. Although the incidence of vaginitis in the 41–50 and > 50 age groups was significantly lower than that of women of childbearing age, they cannot be ignored. The cause of vaginitis should be actively removed, and the primary disease should be treated. The appropriate treatment should be performed according to the vaginal secretion detection results to recover vaginal microecology balance and improve the vaginal resistance to pathogens.

Vaginal inflammation is related to ovarian function and pathogen invasion. During vaginitis, the pathogens consume the glycogen of the epithelial cells, hindering the fermentation of *Bacillus vaginalis* [31, 32]. With increased vaginal pH, the beneficial vaginal bacteria gradually reduce or disappear, releasing ecological niches for colonization and propagation of the pathogens. The colonization of the vagina by pathogens will lead to inflammation aggravation. In this study, only 1.49% of the patients with grade II inflammation had detectable pathogens. The patients with a single pathogen detected mostly had grade III inflammation (84.07%), while the patients with mixed infection mostly had grade IV inflammation (54.95%). In patients with BV, a Nugent score of 9–10 (indicating mostly abnormal flora) was associated with mixed infection [33], indirectly supporting the present study. Another study showed that women with BV and an intermediate Nugent score were associated with more important vaginal exfoliation [34]. Hence, these results suggest that patients presenting with vaginal inflammation grade III-IV have a high probability of vaginal infection, suggesting that empirical treatments could be started pending the microbiological results revealing the exact species involved. The presence of grade IV inflammation suggests the risk of mixed infection, suggesting the empirical use of antibacterial and antifungal treatments pending results. Still, a formal diagnostic study would be required to calculate the sensitivity, specificity, accuracy, and predictive values of the vaginal inflammation grade for infections. The vaginal inflammation grade requires the examination of only a few slides. It can be performed quickly, even before the patient leaves, if necessary, which would speed up the management of the patient.

From a clinical point of view, the results highlight that the incidence of vaginitis with mixed infection is high in women. The presence of BV infection, either alone or in combination with other pathogenic bacteria, should be actively treated with the appropriate antibiotics. Vaginitis is mainly found in women of childbearing age. Therefore, it is necessary to examine the vaginal secretions in this age group when women complain of vulvovaginal discomfort or symptoms to avoid gynecological complications. Some women with grade II vaginal inflammation (considered normal) were detected with pathogens, suggesting that symptoms and possible pathogens should be investigated even for women with mild symptoms. Nevertheless, most women with grade II inflammation had no pathogen detected to explain their abnormal vaginal secretions. In such cases, the cause of the abnormal secretions should be investigated and include non-classical microorganisms causing vaginitis or physical/chemical causes like too tight clothes, soap, perfumes, etc. Furthermore, some women with grade III-IV inflammation had no pathogens detected, indicating that inflammation alone cannot be used to diagnose vaginitis. This study could not collect data regarding treatments, changes in hygiene or sexual practice, follow-up, or treatment outcomes. Still, the treatment should be tailored according to the final diagnosis and the bacteria detected.

There are some limitations to this study. First, we did not detect chlamydia, mycoplasma, and other pathogens, which might affect the results of the pathogen detection rate and mixed infection rate. Second, it was a retrospective study based on an outpatient clinic. We could not collect the pregnancy history, contraceptive measures, and sex life history of the patients because of incomplete available information, resulting in the incomplete baseline data analysis of the patients. Besides, the socioeconomic characteristics could not be found in the medical charts. In addition, the study period was in 2013. Third, the diagnoses were based on the hospital’s routine practice, which does not include 16S ribosomal RNA sequencing, Gram staining, or using the Nugent score. Fourth, the examination report can only list a single diagnosis and not provide more information on mixed infections. Finally, treatment data and treatment outcomes could not be determined. Further multicenter studies are needed in the future.

## Conclusions

In conclusion, about half of Chinese women with abnormal vaginal secretions are positive for pathogens in the study period, indicating pathogens might be prevalent in Chinese women. In addition, patients age and inflammation grade might be related to infection or co-infection. Nevertheless, a multicenter, prospective study with a large sample size should be performed for associations among vaginitis type, infection or co-infection, inflammation grade, and women’s age.

## Data Availability

All data generated or analyzed during this study are included in this published article.

## Abbreviations

BV: Bacterial vaginosis
VVC: Vulvovaginal candidiasis
AV: Aerobic vaginitis
TV: *Trichomonas* vaginitis
CV: Cytolytic vaginosis
DV: Desquamative vaginitis
LE: Leukocyte esterase
HPF: High power field
